# Low-dose CT for lung cancer screening: position paper from the Italian college of thoracic radiology

**DOI:** 10.1007/s11547-022-01471-y

**Published:** 2022-03-20

**Authors:** Mario Silva, Giulia Picozzi, Nicola Sverzellati, Sandra Anglesio, Maurizio Bartolucci, Edoardo Cavigli, Annalisa Deliperi, Massimo Falchini, Fabio Falaschi, Domenico Ghio, Paola Gollini, Anna Rita Larici, Alfonso V. Marchianò, Stefano Palmucci, Lorenzo Preda, Chiara Romei, Carlo Tessa, Cristiano Rampinelli, Mario Mascalchi

**Affiliations:** 1 Department of Medicine and Surgery (DiMeC), University of Parma, Via Gramsci 14, Parma, Italy; 2 Unit of “Scienze Radiologiche”, University Hospital of Parma, Pad. Barbieri, Via Gramsci 14, 43126 Parma, Italy; 3 Istituto Di Studio Prevenzione E Rete Oncologica, Firenze, Italy; 4 Ospedale S. Giovanni Bosco, Torino, Italy; 5 Ospedale Prato, Prato, Italy; 6 AOU Careggi, Firenze, Italy; 7 AOU Pisana, Pisa, Italy; 8 Ospedale Torregalli, Firenze, Italy; 9 Mini-Hospital Sandro Pertini, Capannoli (Pisa), Italy; 10 IRCCS San Raffaele Scientific Institute, Milan, Italy; 11 Dipartimento Di Diagnostica Per Immagini, Radioterapia Oncologica ed Ematologia, Fondazione Policlinico Universitario “A. Gemelli” IRCCS, Università Cattolica del Sacro Cuore Di Roma, Roma, Italy; 12 Department of Radiology, Fondazione IRCCS Istituto Nazionale Dei Tumori, Milan, MI Italy; 13 UOC Radiologia 1, Dipartimento Scienze Mediche Chirurgiche E Tecnologie Avanzate “GF Ingrassia”, Università Di Catania, AOU Policlinico “G. Rodolico-San Marco”, Catania, Italy; 14 IRCCS Fondazione Policlinico San Matteo, Pavia, Italy; 15 Dipartimento Di Scienze Clinico-Chirurgiche, Diagnostiche E Pediatriche, Università Degli Studi Di Pavia, Pavia, Italy; 16 Radiologia Apuane E Lunigiana, Azienda USL Toscana Nord Ovest, Pisa, Italy; 17 Istituto Europeo Di Oncologia, Milano, Italy; 18 Università Di Firenze, Firenze, Italy

**Keywords:** Lung cancer, Lung nodule, Early diagnosis, Screening, Computed tomography, Computer assisted diagnosis

## Abstract

Smoking is the main risk factor for lung cancer (LC), which is the leading cause of cancer-related death worldwide. Independent randomized controlled trials, governmental and inter-governmental task forces, and meta-analyses established that LC screening (LCS) with chest low dose computed tomography (LDCT) decreases the mortality of LC in smokers and former smokers, compared to no-screening, especially in women. Accordingly, several Italian initiatives are offering LCS by LDCT and smoking cessation to about 10,000 high-risk subjects, supported by Private or Public Health Institutions, envisaging a possible population-based screening program. Because LDCT is the backbone of LCS, Italian radiologists with LCS expertise are presenting this position paper that encompasses recommendations for LDCT scan protocol and its reading. Moreover, fundamentals for classification of lung nodules and other findings at LDCT test are detailed along with international guidelines, from the European Society of Thoracic Imaging, the British Thoracic Society, and the American College of Radiology, for their reporting and management in LCS. The Italian College of Thoracic Radiologists produced this document to provide the basics for radiologists who plan to set up or to be involved in LCS, thus fostering homogenous evidence-based approach to the LDCT test over the Italian territory and warrant comparison and analyses throughout National and International practices.

## Introduction

Lung cancer (LC) is the leading cause of cancer-related death in Italy and worldwide, with over 2 millions cases in 2018 [1]. LC is associated with smoking, it is usually diagnosed in advanced stage in variable association with clinical symptoms and has 85% five-year mortality [1]. In Italy, LC is the third most common neoplasm (11% of alla cancers in 2018) while ranking first cause of cancer death (20% of all cancer deaths in 2018) [2].

Smoking cessation is the intervention for primary prevention of LC [3]. It is estimated that 85–90% of LC are associated with cigarette smoking in Italy, where prevalence of smoking people is 23% [2]. Smoking cessation decreases LC risk after 10 years since quitting [4]. Age is the second established risk factor for LC, and environmental and professional exposures represent additional risk factors [5–10].

Screening with low-dose computed tomography (LDCT) is the main intervention for secondary prevention of LC and decreases the LC mortality by 20–30%, especially in women [11–17]. A number of Italian trials contributed to the screening literature. In particular, over 8,000 subjects were enrolled in the three randomized controlled trials that took place in Italy since the early 2000^S^. The Multicentric Italian Lung Detection (MILD, 4,099 participants) showed a statistically significant 39% reduction of lung cancer mortality after 10 years of screening, whereas the ITALUNG Trial (3,206 participants) and the Detection And screening of early lung cancer with Novel imaging Technology (DANTE, 2,450 participants) provided similar results in shorter screening periods and with lower statistical power. Non-randomized trials, including COSMOS and BioMILD in Milan, recruited about 10,000 participants [18, 19], and the bioMILD study (4,119 participants) prospectively investigated the integration of LDCT and blood biomarkers for optimized prolonged screening interval at 3 years [20]. An European Committee for Health Technology Assessment concluded that “screening for lung cancer with LDCT may have a mortality benefit” [21] and the United Stated Preventive Services Task Force (USPSTF) has recently broaden inclusion criteria for LDCT screening [5]. Although COVID-19 infection slowed cancer screening interventions [22], an Italian study demonstrated that LDCT screening can be safely performed during the COVID-19 pandemic [23]. The Cochrane Database of Systematic Reviews is encouraging pilot studies, notably with a short list of necessary outcome measures for continuous quality assurance in data collection and future meta-analyses [24] (Table 1).

**Table 1:** Summary list of outcomes reported according to their order of priority by the Cochrane Database of Systematic Reviews

Summary list of outcomes
Lung cancer related mortality
All-cause mortality
*Incidence of lung cancer* During screening period Post screening period
Recall rates
Harms of screening including the number of invasive tests performed in those with a false positive diagnosis
Impact on smoking behaviour (e.g. cessation, relapse rates, smoking intensity)
Health-related quality of life and/or psychosocial consequences

In 2015, LCS by LDCT started in the USA and is reimbursed by Medicare. In Europe, despite availability of several shared guidelines [25–27], population LCS has not started yet. In Italy, an ongoing discussion is supposed to prepare for inclusion of LCS by LDCT into the governmental healthcare supply (e.g. Livelli Essenziali di Assistenza, LEA), which already includes other population-based cancer screening (e.g. breast, colon-rectum and uterine cervix). These screening interventions are part of the National Prevention Plan, which has purportedly to include high-risk and socially or economically disadvantaged individuals [28].

Several initiatives and studies of LDCT for LCS have been funded and have already started or are next to start in Italy in 2021–2022 (Table 2). They feature some differences in method, thus implying heterogeneity of the proposed screening models, including enrolment criteria and modalities, annual or biennial frequency, strategies to promote smoking cessation, and use of biomarkers. However, taking into account the fundamental role of the radiologist in LCS, we trust sharing fundamentals of radiology practice in LCS is mandatory to warrant quality assurance and allow comparison and/or meta-analyses for informing the next step towards population-based LCS. According to such an objective of harmonization, the Italian College of Thoracic Radiologists discussed and elaborated the present document, which aims to be a practical support for radiologists involved or approaching LCS with LDCT in Italy. The following paragraphs present the current standard of reference for LDCT scan protocol, reading method, classification of findings, reporting and major areas of further management.

**Table 2:** Funded studies of lung cancer screening with low-dose CT in Italy until 10 September 2021

Project	Site(s)	Target sample	Inclusion criteria
Rete Italiana Screening Polmonare (RISP)	Istituto Nazionale Tumori di Milano (and associate centres)	6,500	Age: 55–75 years Smoking history: ≥ 30 pack/years, quit ≤ 10 years
Progetto Ministeriale PEOPLHE	University Hospitals of Parma, Pavia, and Catania	1,500	Age: 50–75 years Smoking history: ≥ 15 cig/day for ≥ 25 years ≥ 10 cig/day for ≥ 30 years quit ≤ 10 years
Italung 2	Florence, Pisa, Massa Carrara	700	Age: 55–75 years Smoking history: ≥ 30 pack/years, quit ≤ 10 years
CCM	Florence, Pisa, Turin, San Raffaele Hospital in Milan	570	Age: 55–75 years Smoking history: ≥ 30 pack/years, quit ≤ 10 years

## Low-dose CT for lung cancer screening: hardware and scan protocol

The screening LDCT is a simple and fast chest examination, which does not require administration of contrast agent. The technical details about hardware, acquisition, and reconstruction of LDCT for LCS were indicated by an expert panel of chest radiologists from the European Society of Thoracic Imaging (ESTI, link to online resource) [29]. Routine LDCT for LCS is hereafter described.

The acquisition starts with a bidimensional scout scan over the chest and is followed by a volume acquisition from apex to lung bases. Deep inspiratory breathhold is mandatory. Any external object must be removed from the chest to minimize radiation exposure and avoid artefacts that would impair nodule measurements. For the volume acquisition, the tube setting is set at low current (typically below 40 mAs) and 120 kVp (or 140) voltage. It is of paramount importance that the tube voltage (and acquisition and reconstruction parameters) is kept consistent through subsequent LDCT examinations of the same subject to allow reliable evaluation of nodule features, especially for subsolid nodules. The above setting aims to minimize the radiation exposure, while maintaining appropriate image quality for volume segmentation [30]. The reduction of radiation exposure is also available via filtering the X-ray beam by tin filter installed between the tube and the aluminium bowtie filter, his technique was proposed by voltage 100 kVp and current 100 mAs with automatic exposure control [31, 32].

The slice collimation must be thin (≤ 1 mm) to grant optimal data quality for image reconstruction. The number of detector rows of the spiral CT scanner is not per se a limitation for LCS. However, CT scanners with few (e.g. 4–16) rows of detectors are becoming obsolete for the purpose of LCS, since they typically require higher radiation doses owing to lower efficiency of old detector technology. Therefore, although screening LDCT has been performed also with few-row CT scanners, the technological development justifies recommendations for up-to-date (64-row or higher) CT scanners. Software development also assists in dose reduction, namely by controlling the noise: iterative reconstructions or deep learning algorithms outstand the old filtered back projection. Phantom studies indicate that advanced reconstruction algorithms together with careful tube setting allow radiation exposures similar to that of chest X-ray (also known as ultra-low dose CT), while being far more sensitive to lung nodules detection [33].

The radiation dose of a screening chest LDCT varies depending on the biometric features (height and weight) of the subject. The ideal threshold of volume computed tomography (CT) dose index (CTDI_vol_) is set below 2.0 mGy by the America College of Radiology. It is noteworthy that the Italian law (number 101 released 31 July 2020) requires to report the radiation exposure associated with every examination on each radiology report. While setting an optimized LDCT protocol for LCS, the radiologist should also bear in mind that the image quality should also allow assessment of pulmonary emphysema and coronary artery calcifications [34], which along with pulmonary nodule represent the so-called BIG-3 [35].

## LDCT test reading

The time required for reading a screening LDCT examination by an experienced radiologist is generally below 10 min, but it can be less than 5 min in case of a negative test [36]. Usually, the LDCT is read by two independent radiologists. This method is similar to that recommended in breast screening and undoubtedly increases the costs of LC (along with the need of dedicated CT scanner spaces and of acquisition and maintenance of software for volumetric assessment of lung nodules size). Not surprisingly, great attention is paid to the possible implementation of Computer Assisted Diagnosis (CAD) systems. In a study in the Netherlands, LDCT reading performed by a single radiologist supported by CAD system replaced double reading [15]. The time required for LCS test reporting varies according to the use of CAD as support to the reader and type of report (free or structured). Adoption of a certified CAD is suggested to help reducing variability in detection rate between readers, while classification might still vary substantially depending on the manual correction often required for nodule segmentation [37]. Overall, the use of CAD for reading LDCT in LCS can be endorsed, but requires an appropriate education on its function, strength, and limits, that have been outlined in specific recommendations by the ESTI [38]. Webinars on the theoretical backgrounds of CAD and practical hands-on workshops by the same expert panel are available to promote CAD use in LCS with LDCT [39]. Nonetheless, the LDCT for LCS should be read by radiologists educated in lung imaging and with specific skills in LCS. For this purpose, since 2019 the ESTI is providing certification courses for theoretical and practical education of radiologists to the practice of LCS [39].

## Test outcome

### Nodule density

The test outcome depends on the presence of non-calcified lung nodules and on their density and size, as well as their possible growth over time. Non-calcified nodules are classified based on their density in solid (homogeneous soft tissue attenuation), non-solid (ground glass opacity: hazy increased attenuation in the lung that does not obliterate the bronchial and vascular margins) or part-solid (mixed non-solid nodule with soft-tissue attenuation components) [40]. Size measurements are meant for solid nodules and solid component of mixed nodules, which substantially drive the test outcome: the greater the size of the solid component, the higher the probability of cancer.

### Nodule size

**Fig. 1:**
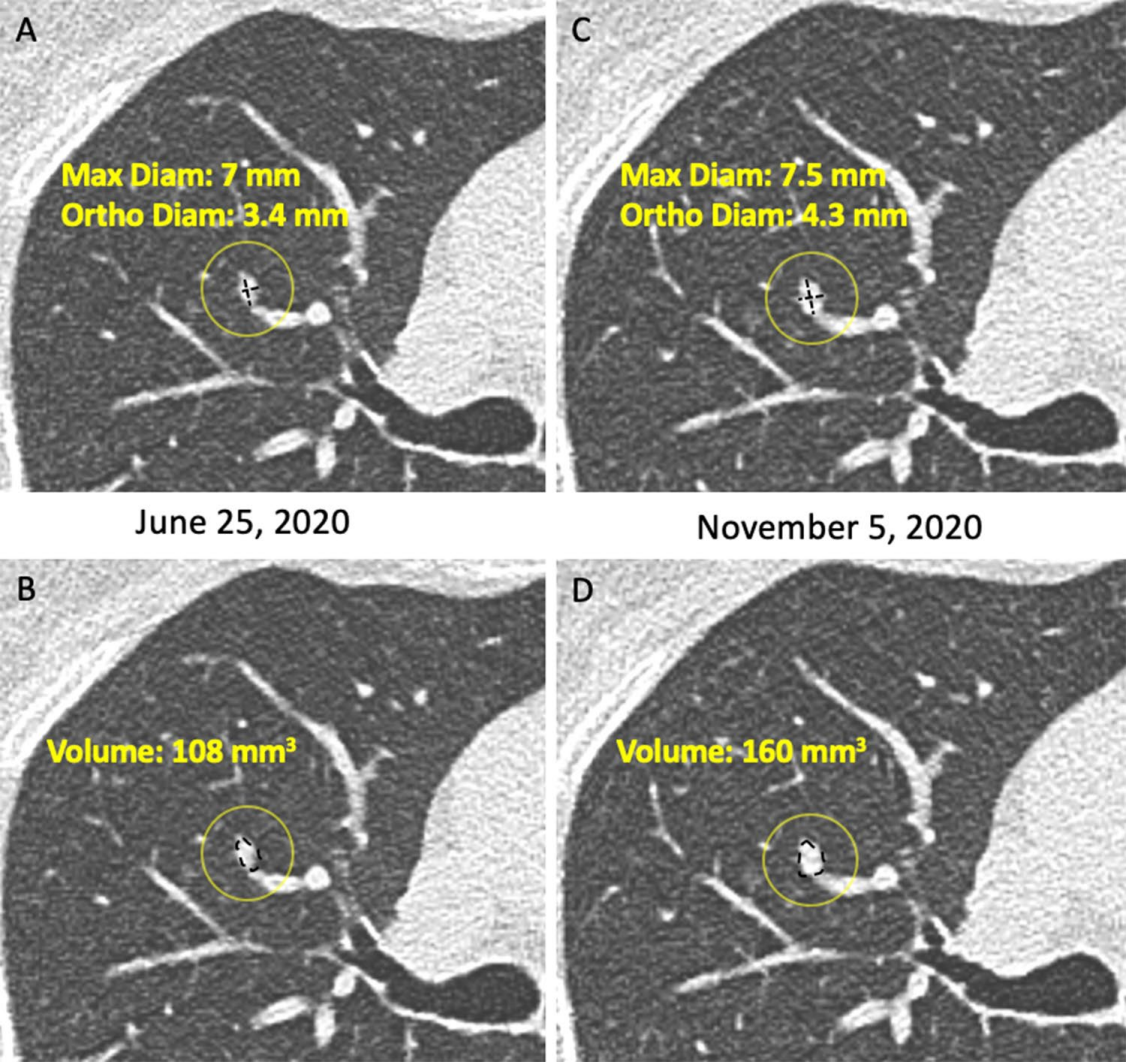
**A**–**D**. Measurement of a solid nodule with histologic diagnosis of adenocarcinoma in the right upper lobe and its growth. Axial CT image showing a solid nodule in the right upper lobe. Two examples of measurement are displayed: **A** manual caliper (maximum diameter 7 mm, orthogonal diameter 3.4 mm, mean diameter 5.2 mm) and **B** semi-automatic volume segmentation (**B**: 108 mm^3^). The follow-up scan shows growth of the solid nodule compared to first detection, which is below the minimum threshold of 2 mm by manual caliper (**C**: 7.5 × 4.3 mm, mean diameter 5.9 mm) and above the minimum threshold of 25% by volume segmentation (160 mm^3^): such discrepancy reflects into divergent classification as stable by manual caliper and grown by volume segmentation, for this solid nodule that was diagnosed adenocarcinoma. Furthermore, the longitudinal calculation of growth rate shows different estimate of volume doubling time by manual caliper (445 days) or volume segmentation (236 days)

**Fig. 2:**
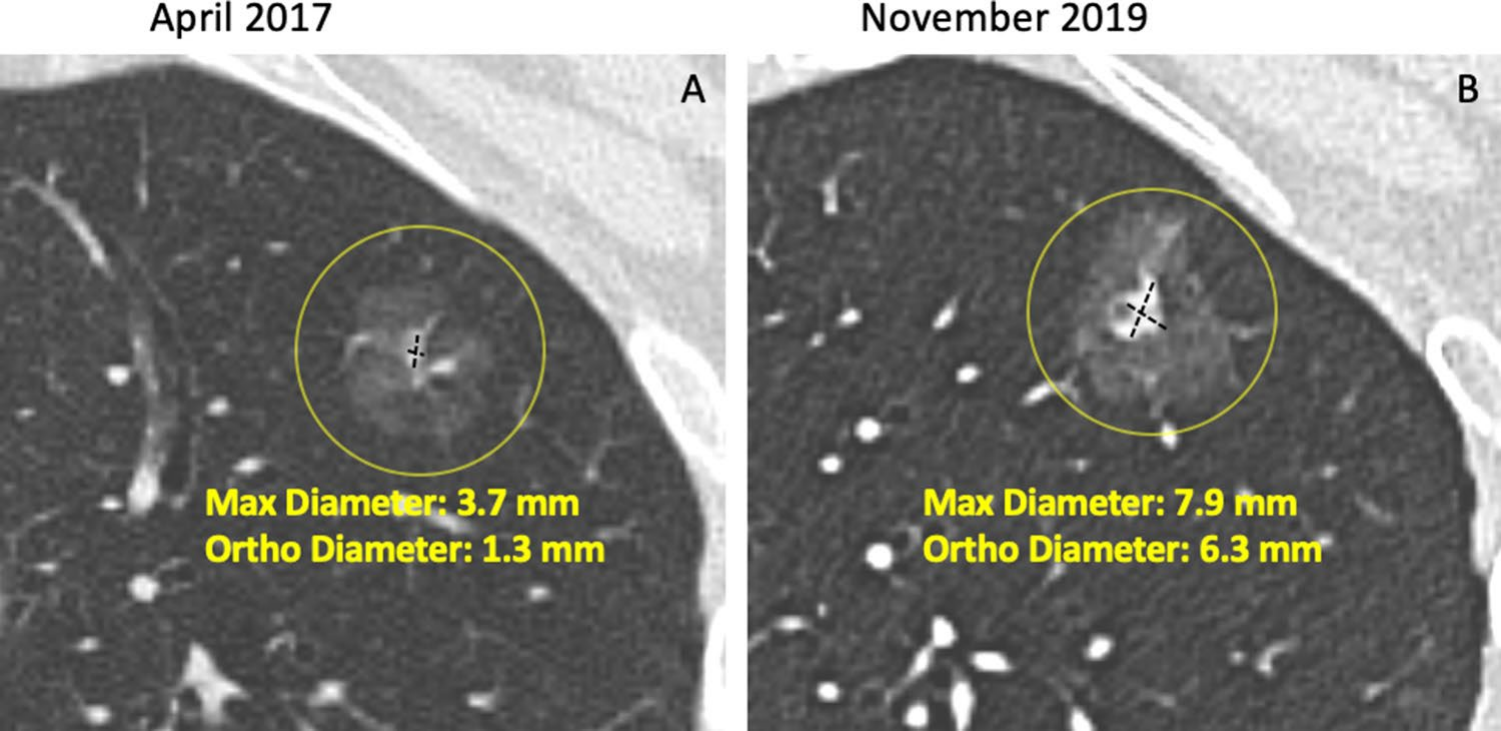
**A**–**B**. Measurement of a part-solid nodule in the left upper lobe and its growth. Axial CT image showing a part-solid nodule in the left upper lobe. The size of the solid component by manual caliper at first detection (A: maximum diameter 3.7 mm, orthogonal diameter 1.3 mm, mean diameter 2.5 mm) is thereafter confidently increased at follow up scan (**B**: 7.9 × 6.3 mm, mean diameter 7.1 mm). The variable and limited density difference between solid component and non-solid component represents a factor for variability of semi-automated volume segmentation. Moreover, the figure shows small vessels abutting the surface of the solid component, that is one common factor that further hampers the use of volume segmentation of solid core in part-solid nodules

**Fig. 3:**
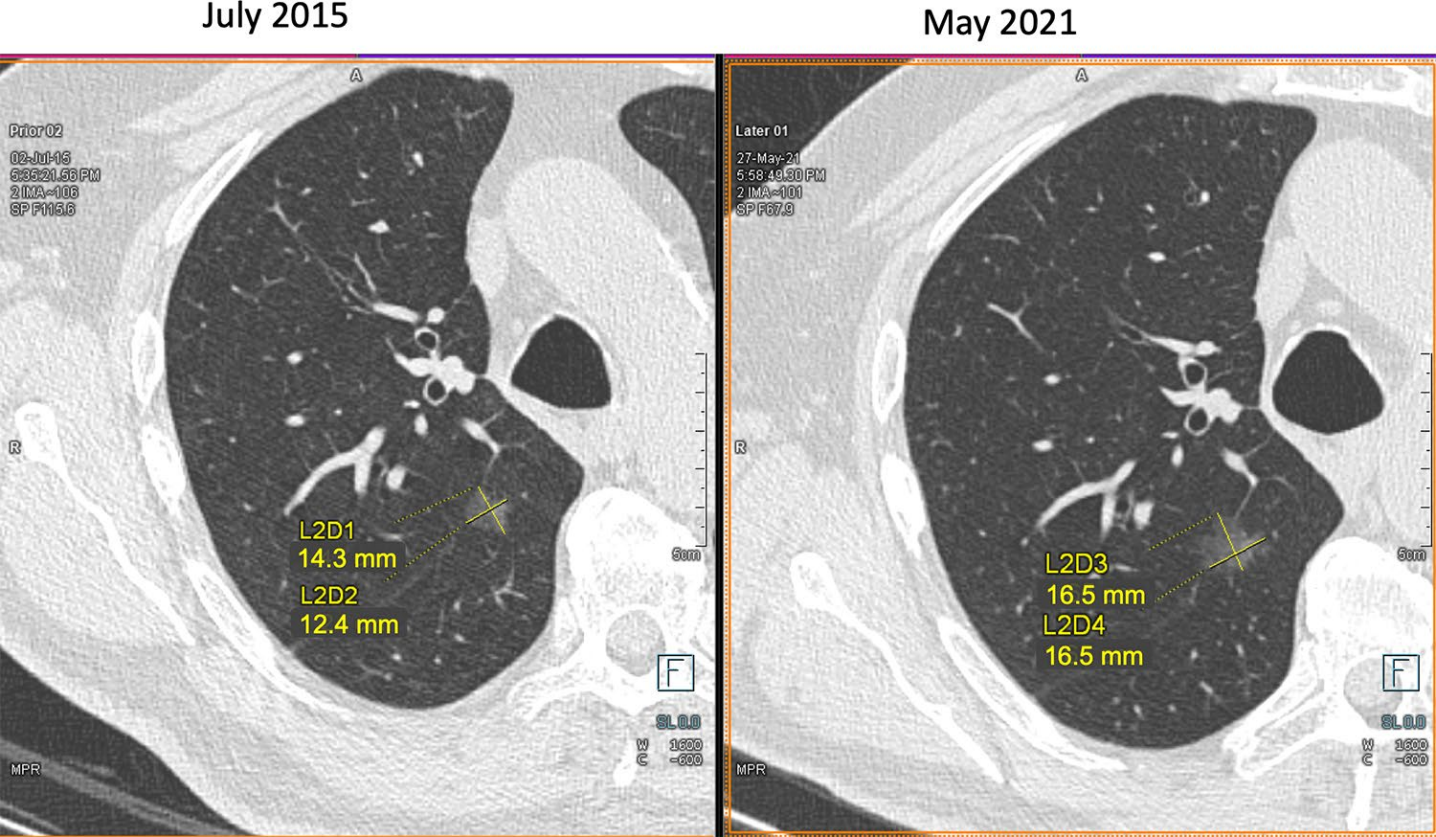
**A**–**B**. Measurement of a non-solid nodule (ground glass opacity) in the apical segment of the right lower lobe and its growth. Axial CT image showing a non-solid nodule in apical segment of the right lower lobe. The measurement by manual caliper at first detection (maximum diameter 14.3 mm, orthogonal diameter 12.4 mm, mean diameter 13.4 mm) and follow up scan (**B**: 16.5 × 16.5 mm, mean diameter 16.5 mm). The variable and limited density difference between the non-solid nodule and the surrounding parenchyma represents a factor for variability of semi-automated volume segmentation

The size of non-calcified solid nodules or of solid component of a mixed nodule can be assessed using bidimensional manual measurement (maximum and orthogonal diameter for calculation of the mean diameter, to one decimal point) [11] or volumetric measurement aided by software [15] (Figs. 1, 2, 3).

Volume measurement of solid nodule is more reproducible and sensitive to nodule growth than manual caliper (Fig. 1). Volumetric software was adopted in the NELSON trial [15] and is strongly recommended in Europe [25]. However, volumetry has a limitations in certain scenarios in which bidimensional (e.g. mean diameter or longest diameter) measurements should be preferred. These scenarios include: (1) solid nodules abutting solid surfaces such as pleura or vessel; (2) solid component of part-solid nodule; (3) non-solid nodules (Fig. 3).

### Software for nodule volumetry

Several types of software for nodule segmentation and volume estimation are commercially available. Their performance is much variable with possible over or under-estimation of the nodule size [41]. This variability may impact recall and detection rates even for different releases of the same software. A consistent use of the same software type and release is therefore recommended over time, especially when different software and releases may be available on different CT scanners in the active screening centres.

### Classification of the test results

The Lung-RADS 1.1 system is recommended for classification of findings of the chest LDCT for LCS (link to online resource) [42, 43]. It warrants shared lexicon and clear report interpretation. It provides a validated protocol for diagnostic work-up and incorporates the most recent advances in knowledge (e.g. peri-fissural nodules, non-solid nodules, and in the next 2.0 release an update is anticipated dealing with classification and management of cystic lesions [44]. Noteworthy, this reporting system has been adopted in the largest LCS practice, which started in 2015 in the USA and currently involves over a million participants [45]. There is evidence that use of Lung-RADS decreases the rate of false-positive results in lung cancer screening [46].

According to Lung-RADS 1.1 system, lung nodules found on LDCT are divided in 6 categories from 1 to 4X based on density and size characteristics and on the evidence of growth [42]. The higher the category, the higher the risk of malignancy of a given nodule. The category of each LDCT test result should be coded according to the nodule with the highest degree of suspicion, namely the nodule with the highest score (also known as the “dominant nodule”). Lung-RADS 1.1 indicates the size threshold for lung nodule reporting mean diameter:

$$\frac{{{\text{maximum}}\;{\text{ diameter}} + {\text{ orthogonal}}\;{\text{ diameter}}}}{2}$$

approximated to one decimal point, and it introduced the use of volume.

Each category has a different management. Nodules belonging to category 1 and 2 correspond to a negative screening test, for which a scheduled annual LDCT is recommended by ACR. Nonetheless, biennial LDCT screening for category 1 and 2 is gaining more and more consensus along with integrated risk models, and this might become the preferred option to optimize cost–benefit ratio of LCS [47, 48]. Differently, category 3 (probably benign), category 4A (suspicious) and category 4B or 4X (very suspicious) nodules qualify for a non-negative screening test. According to Lung-RADS 1.1 system, category 3 lung nodules require 6-month follow-up LDCT, category 4A lung nodules require 3-months follow-up LDCT to ascertain size evolution over time, whereas category 4B and 4X lung nodules require immediate work-up (see below). For new large nodules appearing on a scheduled LDCT screening round and matching 4B category, a 1-month follow-up LDCT is recommended after antibiotic therapy to ascertain potential infectious or inflammatory conditions.

The inclusion of a “S” label to nodule categories allows to indicate other clinically significant or potentially clinically significant findings different from lung cancer [49, 50].

For category 3 (probably benign) nodules Lung-RADS 1.1 recommends a 6 month follow-up LDCT. This 6-month interval is a matter of debate, since other guidelines suggest a shorter 3-month control for the management of the so-called indeterminate nodule [25, 51]. A 3-month follow-up might help contain anxiety for indeterminate results and be more conservative. However, a shorter interval is associated with higher risk that bidimensional but also volume size changes are small and inconclusive. This concept was recently forced and stretched further by the need of delaying LCS screening activity during the pandemic from the severe acute respiratory syndrome coronavirus 2 (SARS-CoV-2). LCS participants with longer follow-up LDCT for “indeterminate nodule” did not incur in stage shift at the time of lung cancer diagnosis [52].

The size threshold for category 4A (suspicious) solid nodules is 8 mm in mean diameter and 268 mm^3^ in volume on baseline LDCT and 6 mm and 113 mm^3^ for new nodules appearing at annual repeat LDCT. For category 4B (very suspicious) nodules it is encouraged to use the Brock algorithm that incorporates non-radiological features to predict nodule malignancy [53].

In case of non-solid nodules, Lung-RADS 1.1 recommends follow-up LDCT or interventions only for nodules with a mean diameter ≥ 3 cm, but also in this case the debate is still open [54].

The 4X (very suspicious) category that requires immediate diagnostic work-up can be assigned to small nodules based on special suspicious features as evidence of nodular spiculations or associated mediastinal lymphoadenomegaly [55]. Also a non-solid nodule below 3 cm in size (category 2) should be upgraded to 4X if any interval growth is measured.

### Structured report

Structured report is encouraged to standardize and make LCS with LDCT consistent at large. The ESTI proposed a simplified model for structured report of LDCT [56], which includes the necessary features for nodule descriptions in the LCS setting (Table 3).

**Table 3:**
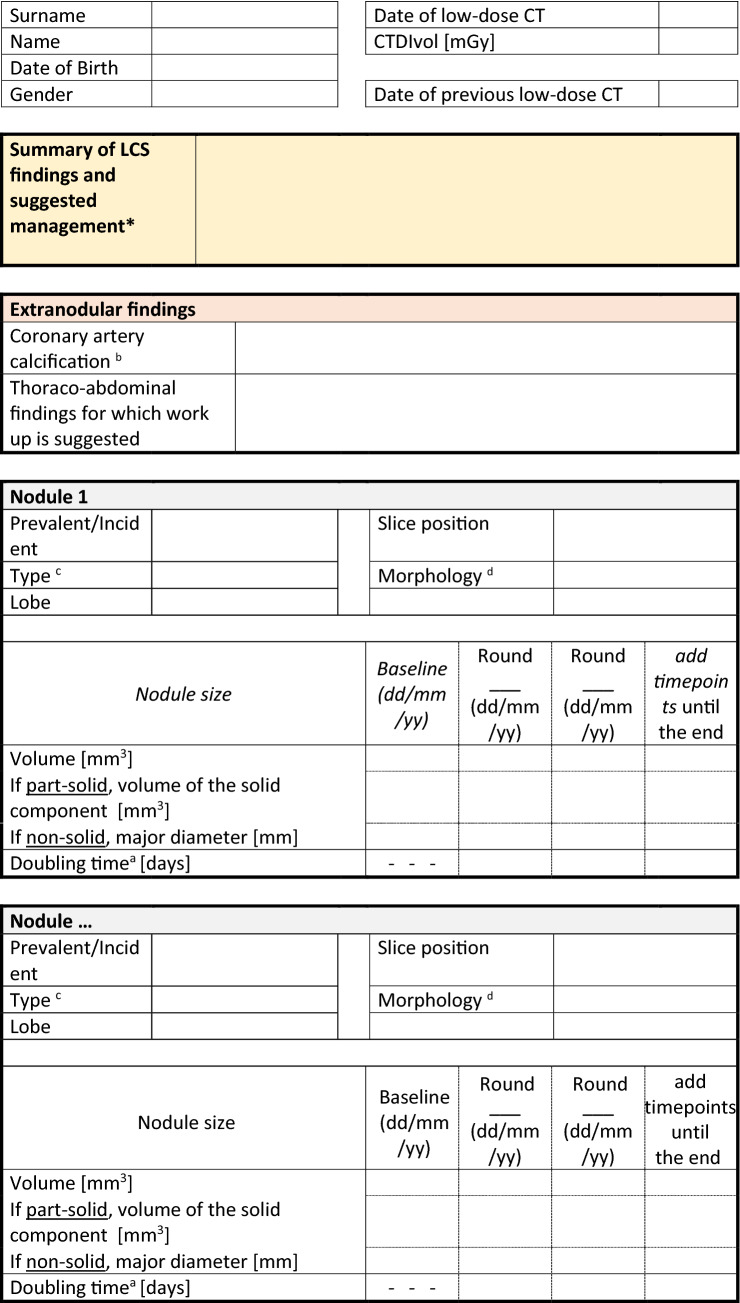
Structured report for LDCT in LCS ( modified from www.esti.org) with links for computation of the risk of malignancy of a nodule at baseline (Brock methods)* and of the growth at subsequent low-dose CT examinations^a^

*The Brock model calculator is available online from several certified resources, for instance the “PN Risk Calculator” form the British Thoracic Society, either diameter or volume can be used (https://www.brit-thoracic.org.uk/quality-improvement/guidelines/pulmonary-nodules/pn-risk-calculator/)

^a^The volume doubling time (VDT) can be calculated with measurement of nodule volume or bit is also accepted by geometric translation of mean diameter. Noteworthy, the VDT is accepted for the specific characterization of solid nodule. The VDT is currently provided by most CADe/CADx software, moreover it is also found online, for instance the “PN Risk Calculator” della British Thoracic Society.

^b^Coronary artery calcifications can be assessed by semi-quantitative method (0 = absent; 1 = mild; 2 = moderate; 3 = severe) or more complex (from 0 to 12 score) visual scales (see https://doi.org/10.1148/radiol.15142062 and https://doi.org/10.1148/radiol.10100383)

^c^The type of nodule is defined according to its: solid/part-solid/non-solid/calcified

^d^The morphology of nodule is found in the literature and is usually aimed to stratify risk: spiculation, perifissural nodule

The non-negative LDCT test, namely lung nodules matching Lung-RADS 1.1 categories from 2 to 4X, should be discussed in multidisciplinary teams. To contain costs associated with this procedure, it has been suggested to restrict the multidisciplinary team discussion to lung nodules ≥ 200 mm^3^ [57].

## Diagnostic work-up in positive tests

The work-up of suspicious or highly suspicious (Lung-RADS 1.1 categories 4A-4X) nodules with a solid component exceeding 8 mm can be performed with 18F-fluoro-2-deoxy-glucose -positron emission tomography (FDG-PET)/CT, CT-guided fine needle aspiration or core biopsy, and Video Assisted Thoracic Surgery (VATS). The choice of work-up strategy usually reflects local availability and expertise and is usually accompanied by a staging contrast enhanced CT at standard dose.

Differently, follow-up LDCT is the management tool for indeterminate (Lung-RADS 1.1 category 3) nodules at baseline or annual repeat LDCT with the goal to ascertain possible nodule growth. The latter is trusted at ≥ 25% increase of lesion volume [26], or an increase of the mean diameter > 1.5 mm (Fig. 1). Integrated description of growth dynamics is anticipated for the next Lung-RADS 2.0 release [43, 44]. The dynamics of growth in solid lesion can be estimated by serial LDCT and calculation of the Volume Doubling Time (VDT) (Fig. 1). Notably, the VDT can be calculated based on either mean diameter or segmented volume: a VDT ≤ 400 days is associated with a malignant nodules [25] (Fig. 1). So far, the VDT was validated for solid lung nodules, but not for part-solid or non-solid nodules. For the latter two types of nodules, increase of a solid component in mixed nodules or appearance of new solid component in a former non-solid nodule is considered a sign of significant growth and potential malignancy [58] (Fig. 2, 3).

## Collateral and incidental findings

Additional findings in screening LDCT are common, being observed in 4.4 to 40.7% cases. They are more frequent with increasing participants age and can imply further evaluations. The wide variability reflects inconsistent definition of such findings and especially their clinical relevance [59].

Recommendations about reporting of additional findings have evolved [60–62]. To date, the growing experience and advances in knowledge on LDCT screening suggest a “granular interpretation”. The reference guidelines for such a critical interpretation are provided both by the American College of Radiology [50] and the National Health System England [63]. Findings unrelated to LC and pulmonary nodules can be distinguished in smoking-related and non-smoking-related. We shall label smoking-related findings as “collateral findings” which include calcifications of coronary arteries (CAC), pulmonary emphysema and interstitial pulmonary abnormality/disease. The remainder, namely the wide array of non-smoking-related findings will be interpreted as the true “incidental” findings (Table 4).

**Table 4:** Examples of incidental (non-smoking related) extra-thoracic findings in LDCT for LCS, modified from https://www.cancercareontario.ca/en/content/recommendations-management-actionable-incidental-findings-lung-cancer-screening-pilot-people-high-risk

Incidental findings	Not Actionable	Actionable
Thyroid	< 1.5 cm and lack of suspicious features RECOMMENDATION: No further evaluation	≥ 1.5 cm and/or suspicious findings (Abnormal lymph node (calcifications, cystic components) and/or invasion of local tissues by thyroid nodule) RECOMMENDATION: Thyroid Ultrasound
Ascending aorta dilatation	Ascending aorta diameter 4.0–4.5 cm RECOMMENDATION: report measure in body of text and remeasure on annual screening CT	Ascending aorta diameter ≥ 4.5–4.9 cm RECOMMENDATION: Echocardiogram and consider referral to cardiology or cardiac surgery Ascending aorta diameter ≥ 5.0 cm RECOMMENDATION: Echocardiogram and refer to cardiac surgery
Breast nodule or asymmetry	Definitely benign nodules (e.g. lipoma, densely calcified nodules, etc.) RECOMMENDATION: No further evaluation	Indeterminate breast findings (e.g. non-calcified nodules, asymmetries, etc.) RECOMMENDATION: Mammogram
Indeterminate renal nodule or mass	Simple renal cysts (− 10 to 20 HU), cysts > 70 HU, and nodules too small to characterize. Fatty nodules without calcification (angiomyolipomas) RECOMMENDATION: No further evaluation	All other lesions: Defer to judgement of reading radiologist RECOMMENDATION: Ultrasound or additional imaging as per institutional practice
Indeterminate hepatic nodule(s) or mass	Too small to characterize or with benign features (sharply marginated, homogeneous, ≤ 20 HU) RECOMMENDATION: No further evaluation	Suspicious features (ill-defined margins, heterogeneous density, mural thickening or nodularity, thick septa) or with cirrhosis RECOMMENDATION: Ultrasound or additional imaging as per institutional practice

### Collateral findings

**Fig. 4:**
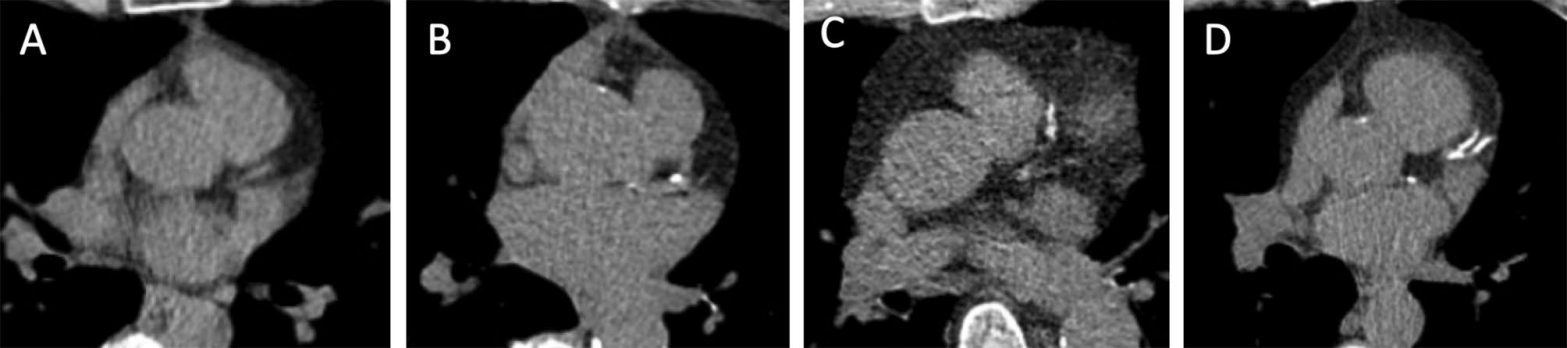
**A**–**D**. Collateral (smoking-related) findings in screening LDCT. Calcifications of the coronary arteries. Axial CT images at the level of the left main coronary artery showing different degrees of coronary artery calcification (CAC): absent = 0 (**A**), mild = 1 (**B**), moderate = 2 (**C**) and severe = 3 (**D**). According to the scale proposed by Chiles et al. [67], isolated flecks correspond to a mild degree (**B**), continuous calcification along the vessel correspond a severe degree (**D**)

**Fig. 5:**
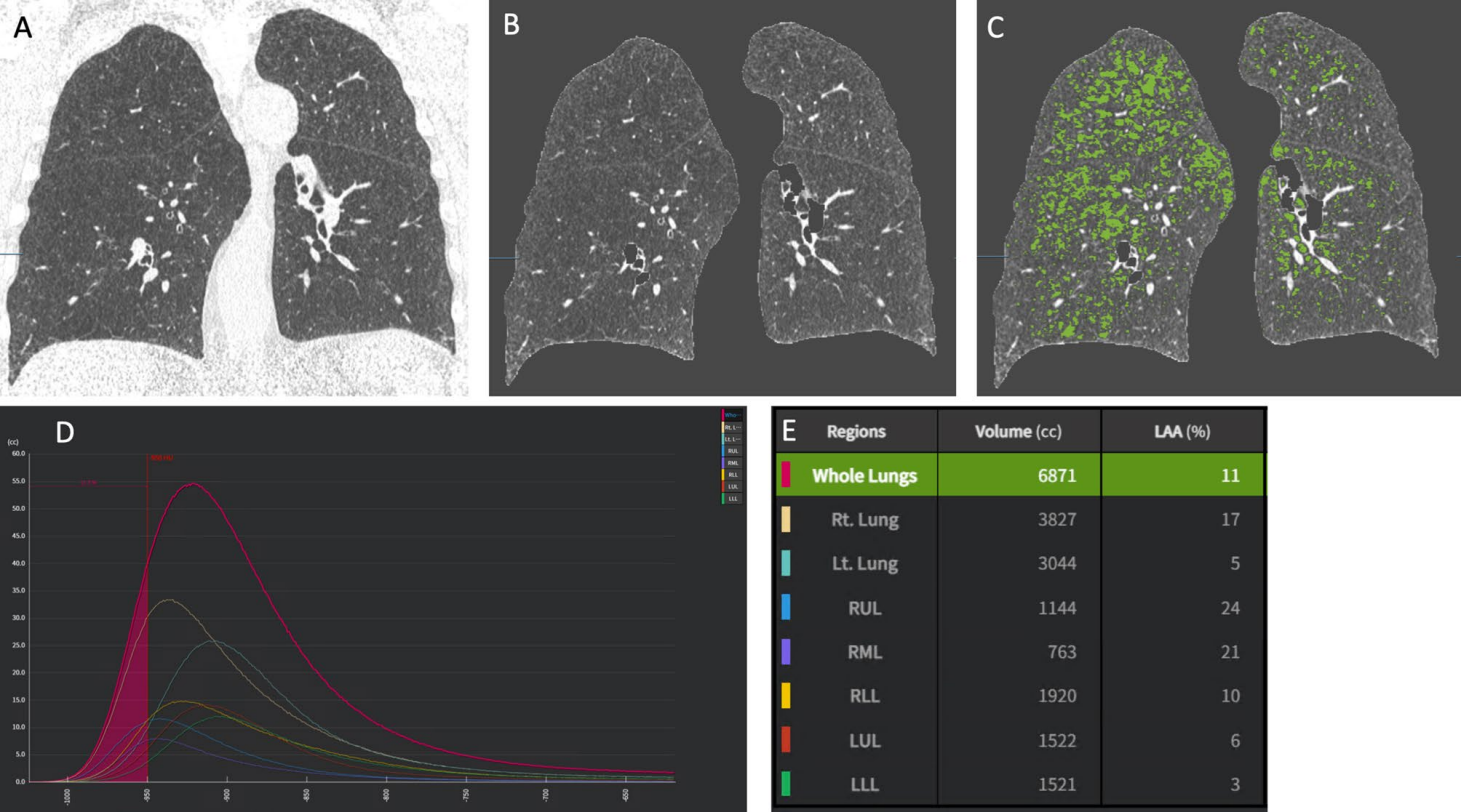
**A**–**D**. Collateral (smoking-related) findings in screening LDCT. Quantification of pulmonary emphysema with application of the 950HU density mask. Pulmonary emphysema quantified by density mask with segmentation of lung areas with density lower than − 950 HU. The example shows the step-wise process of segmentation of lung parenchyma (A: native image; **B**: extraction of lung volume) and subsequent quantitation of emphysema extent represented as low attenuation area (LAA) with density below − 950 HU, as represented by green overlay (**C**). The density histogram (**D**) shows the distribution of density across the lung volume, and allows to quantify the proportion of LAA below -950 HU as relative extent compared to the overall lung volume (**E**), namely 11% in this example (specific lobar quantitation is also provided)

**Fig. 6:**
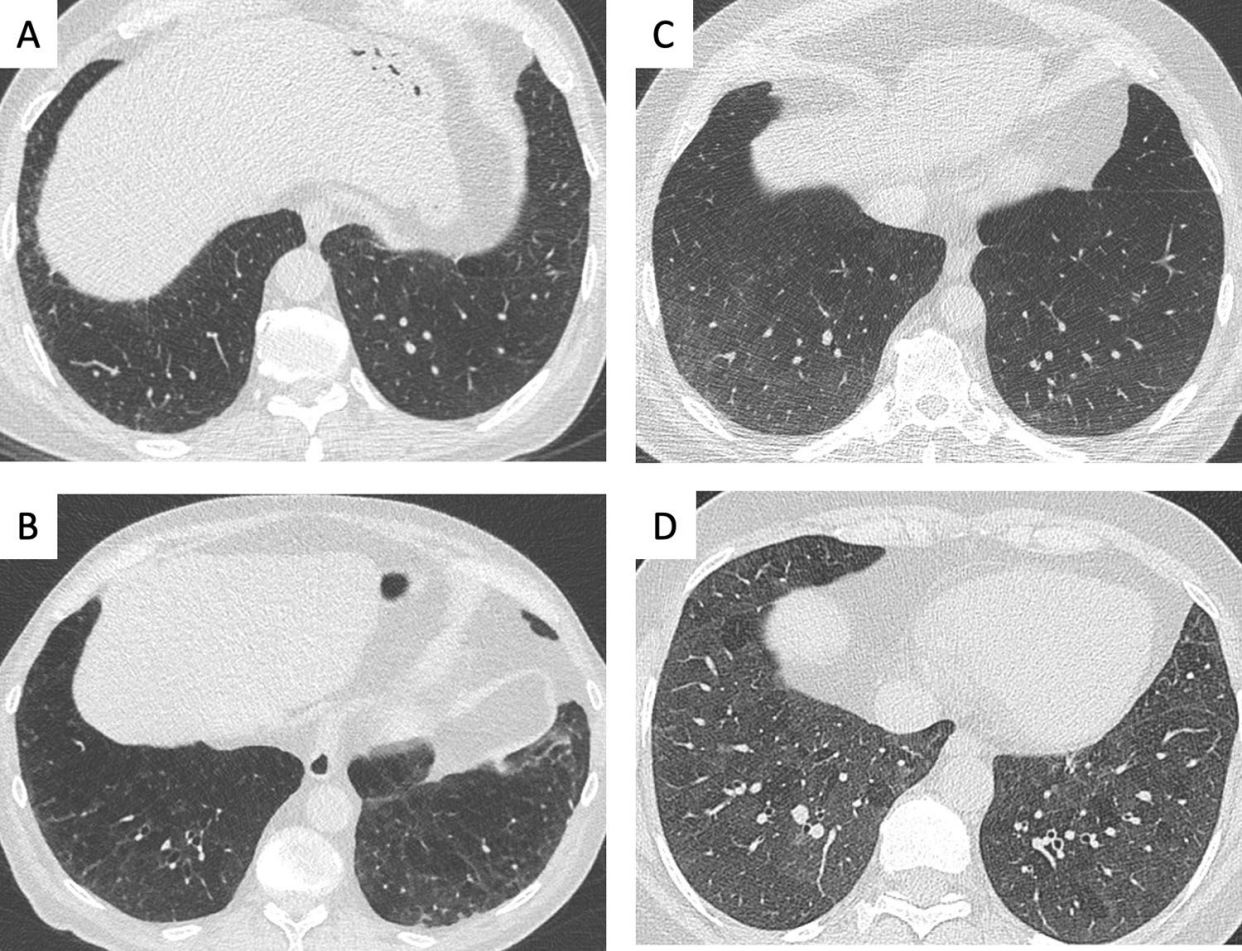
**A**–**D**. Collateral (smoking-related) findings in screening LDCT:interstitial lung abnormalities with varying extent and morphology.Axial CT image at the level of mid-lower chest showing different patterns of interstitial lung abnormalities with varying severity: **A** minor reticulation in right lateral sulcus; **B** reticulation with signs of bronchiolar traction in the lower lobes; **C** ground-glass opacity with mild extent in the lower lobes; **D** ground-glass opacity with extensive distribution in the lower lobes, associated with minimal areas of parenchymal sparing with lobular distribution. These findings variably represent smoking related disease, with either reversible or irreversible behaviour worth of multidisciplinary discussion

Calcifications of coronary arteries, pulmonary emphysema and interstitial pulmonary abnormalities/disease (Figs. 4, 5, 6) have definite importance having a prognostic value in high-risk smokers and former smokers undergoing LCS with LDCT. In fact, they are associated with increased risk of morbidity and mortality [34], being cardiovascular (CV) disease and respiratory diseases the main non-neoplastic causes of death in LCS participants [11, 12].

#### Calcifications of coronary arteries

CAC are recognized as an independent “risk-enhancing factor” for CV disease [64], because their severity is associated with an increased risk of CV events and mortality, both in smokers and former smokers [65–68]. CAC can be assessed by LDCT using visual score of varying complexity or using software [67]. For screening practice, a swift visual score is emerging [68–71]: 0 = absent; 1 = mild; 2 = moderate; 3 = severe (Fig. 4) [67]. When combined with calcification of the aortic valve, severe CAC is associated with further increase of CV death [72].

#### Pulmonary emphysema

Densitometry is more reproducible than visual rating for assessment of pulmonary emphysema and must be preferred also in LCS participants [73]. The assessment of lung density in LDCT examinations for LCS (Fig. 5) is valuable since emphysema severity is correlated with either LC incidence, and hence it adds to the post-test risk of LC [74, 75]. Moreover, lung density is correlated with pulmonary function test, smoking history and smoking-cessation [76], and the overall prognosis [77]. Several densitometric measurements can be used for the definition of emphysema presence and severity [78]. Usually “significant” emphysema is assigned when the Relative Area (RA) or Low Attenuation Area (LAA) ≤ 950 Hounsfield Units exceeds 6% of lung parenchyma [79, 80] (Fig. 5).

#### Interstitial lung disease

Evidence of interstitial lung abnormalities (ILA) or disease (ILD) (Fig. 6) can be particularly relevant in subjects undergoing LCS with LDCT [81]. In fact, ILA can be seen on LDCT in completely asymptomatics subjects [82]. Detection of ILA and its inclusion in the LDCT report with possible discussion in a dedicated multidisciplinary team can add to functional profiling. Identification of ILA should be a strong motivation for quit smoking, whereas the pharmaceutical treatment should be reserved to subjects with established ILD. Moreover, ILA/ILD is a risk factor for also severe complications of LC treatment, including surgery, medical, and radiation therapy.

Admittedly, the ultimate impact of reporting collateral findings in LDCT for LCS and their cost/benefit ratio are hard to define, because the downstream consultation, intervention of primary and secondary prevention and pharmacological treatment have not yet been established. However, certainly, their identification must be used to support smoking quit through access to smoking cessation programs in current smokers who represent the majority of LCS participants. Smoking cessation is offered altogether with LDCT examinations in most LCS initiatives in Italy.

### Incidental findings

Screening LDCT can reveal thoracic and extra-thoracic findings which are unrelated to smoking, and represent true “incidental” findings. A Canadian working group classified incidental findings into non-actionable and actionable (Table 4). Benign non-actionable conditions should not be reported. On the other hand, attention must be paid to detection and reporting of actionable findings as dilatation of the ascending aorta or, especially, those potentially associated with malignant lesions of lymphnodes, thyroid gland, thymus, breast, hepato-pacreatic region, adrenal glands, and the kidneys [83]. Extra-pulmonary cancers were diagnosed in 0.5% of subjects participating to the COSMOS observational study in Milan, Italy and in 0.39% of subjects randomized to LDCT in the NLST trial in US [83, 84]. The American College of Radiology Committee on management of incidental findings in chest CT recommends reporting of mediastinal lymphoadenopathy, mediastinal masses, pericardial abnormalities, dilation of the thoracic aorta and of the pulmonary artery [49].

## LDCT screening interval, duration and personalization

The recommended interval for LDCT screening is once a year [5]. However, several studies demonstrated that two-year interval allows efficient surveillance while reducing radiation exposure and costs [85–87], whereas longer interval is associated with increased number of advanced LC [85]. The possibility of biennial LDCT screening test should be reserved to subjects with negative baseline by LungRADS 1.1, which represent over 70% of LDCT [47].

Screening should start at age 50 and interrupted at 80 [5]. Moreover, LDCT screening is not recommended for former smokers who quit smoking > 15 years or for people not eligible for or not willing to undergo LC surgery.

So far, the selection of subjects at risk of LC has been mainly based on age and smoking history measured by pack-years. However, the yield of these selection criteria seems relatively low compared to multifactorial profiling [19, 88] including pre-test (before LDCT) findings related to personal and family history [53, 89]. Moreover, several multifactorial risk models aim also to post-test risk refinement by inclusion of findings from LDCT [77, 90]. These factors contribute to stratification of LC risk, as well as of CV and respiratory morbidity and mortality that can ultimately hinder possibility of surgical treatment [5].

The above two general considerations underly the quest of LCS personalization [88, 91, 92] both for LDCT interval [75, 93] and LCS duration in the individual lifetime, also considering the theoretical risk from ionizing radiations [94].

## Harms of screening

As for every screening intervention, chest LDCT has its harms [95, 96]. In this manuscript we will mention harms directly related to the radiological practice. They include false-positive results with downstream unnecessary (or even harmful) investigations and invasive work-up, overdiagnosis, distress and anxiety due to indetermined test results, and radiation-induced cancer [97]. In particular, overdiagnosis is a topic of active debate in LCS. Subsolid nodule is probably the most prominent finding associated with slow and potentially clinically indolent growth [58, 98], whose progrostic weight may ultimately be overcome by competing causes of death in heavy smokers or former smokers [77].

On the other hand, also false-negative findings are to be accounted for in LCS practice and this is prone to different interpretations [99]. However, estimate of false-negative rates in an important metric for quality assurance in LCS. False-negative can derive from different moments of the LCS practice, including reading of the LDCT test and definition of nodule management protocols. Accordingly, in the NELSON trial the majority of false-negatives could be attributed to detection or interpretation error [100], whereas the risk of missed cancers increased significantly if screening interval was prolonged at 2.5 years [85].

Psychological distress related to LDCT findings is a further potential harm, which is directly linked to the radiology report [97]. The Danish Lung Cancer Screening Trial investigated the psychological distress in LCS and found that data so far available might be biased by selection of a more robust population [101]. This is probably due to the more favourable socio-demographic profile of people participating in the early LCS trials. Looking towards LCS implementation in the general population, the radiologist should strain towards improved communication of the findings to the screening participant, and this might include accounting for person-hours dedicated to the verbal communication of the report.

## Conclusions

In conclusion, LDCT represents the standard of reference for LCS. The use of LDCT as preferred test in LCS is intended as optimal practice, yet not perfect. The Italian College of Thoracic Radiologists is convinced that the use of quality assurance references is mandatory to make population practice as accurate as LCS trial results. Technology update is mandatory to maintain appropriate quality of LCS practice, while continuous education is warranted to follow the most appropriate evidence, similarly to what was already witnessed in mammography screening. The near future of LDCT for LCS calls for preparedness in technology and medical skills, the next step is eventually foreseen in continuous optimization of resources [102].

## Declarations

### Human or animals rights

No involvement of Participants or Animals.
